# Integration sites of *bla*_CTX−M−1_ relate to IncI1 plasmid phylogeny in *Salmonella* isolates from non-human sources in Germany

**DOI:** 10.3389/fmicb.2025.1711391

**Published:** 2026-01-21

**Authors:** Aitor Atxaerandio-Landa, Maria Borowiak, Angelina Groger, Alexandra Irrgang, Burkhard Malorny, Istvan Szabo, Jennie Fischer

**Affiliations:** 1 MikroIker Research Group, Faculty of Pharmacy, University of the Basque Country UPV/EHU, Vitoria-Gasteiz, Spain; 2 Department of Biological Safety, German Federal Institute for Risk Assessment (BfR), Berlin, Germany

**Keywords:** *Salmonella*, whole genome sequencing (WGS), antimicrobial resistance gene (ARG), plasmids, integration sites, CTX-M-1

## Abstract

Antimicrobial resistance to cephalosporins in *Enterobacterales* is commonly mediated by extended-spectrum β-lactamases (ESBL). The ESBL-encoding gene most frequently detected in *Salmonella* isolates from livestock and the second most frequently detected in *Salmonella* isolates in humans in Germany is the *bla*_CTX−M−1_ gene. In this study, we characterize ESBL-producing *Salmonella enterica* collected from non-human sources in Germany, with a particular focus on *bla*_CTX−M−1_ harboring IncI1 plasmids. Therefore, a total of 95 *bla*_CTX−M−1_ positive isolates (*S*. Derby, *S*. Infantis, and *S*. Typhimurium/1,4,[5],12:i:-) from food and animal origin were investigated using short and long-read Whole-Genome Sequencing (WGS) with subsequent in-depth characterization and phylogenetic analysis of the samples and associated mobile genetic elements. WGS revealed a diverse population of *bla*_CTX−M−1_-producing *S. enterica* isolates in German food and animal samples. In 66 of the 95 isolates, an IncI1 plasmid could be detected. A total of 38 IncI1 positive isolates were selected for long-read sequencing to confirm the location of *bla*_CTX−M−1_ on the IncI plasmid. Additionally, to our 38 *bla*_CTX−M−1_ harboring IncI plasmids, further *bla*_CTX−M−1_ harboring IncI plasmids (*n* = 103) from the Plasmid Library Search Database (PLSDB), derived from different host bacteria, isolation sources, and geographical locations, were analyzed in detail to gain a deeper insight into IncI1 plasmid evolution. Results revealed that the *bla*_CTX−M−1_ gene was associated with the IS*Ecp*1 transposable element in all but two cases. A total of six distinct integration sites (ISts) were detected across 141 IncI1 plasmids studied here. The integration sites correlated with the plasmid ST and the plasmid phylogeny, regardless of the sample origin, host bacterium, or *Salmonella* serovar. In conclusion, the emergence of serovar-specific or geographically restricted CTX-M-1 encoding IncI1 plasmids appears to play a minor role. In contrast, evidence suggests that a few successful IncI1 plasmid lineages/plasmid ST types are the primary vehicles for *bla*_CTX−M−1_ gene transmission in *Salmonella* isolates from diverse geographical origins and sources along the food production chain in Germany.

## Introduction

Salmonellosis caused by non-typhoidal *Salmonella enterica* subsp., *S*. *enterica*, is one of the most common foodborne diseases worldwide (Popa and Popa, 2021) and the leading cause of food-related outbreaks in the European Union (EU; EFSA and ECDC, 2023). Non-typhoidal *Salmonella* are estimated to cause approximately 150 million illnesses and 60,000 deaths worldwide each year (Marchello et al., 2022; Plumb et al., 2023). *Salmonella* naturally inhabits the intestines of livestock (Demirbilek, 2018) and can spread to the food chain through fecal contamination, e.g., during slaughter or food processing (Buncic and Sofos, 2020). *Salmonella* contamination can occur in various types of food, including raw or undercooked meat, eggs, and dairy products (Alberto et al., 2012).

In Germany, the four most commonly reported serovars associated with foodborne salmonellosis in humans over the last years are Infantis, Derby, Typhimurium (including its monophasic variant 1,4,[5],12:i:-), and Enteritidis, the latter two serovars accounting for about 70% of all cases (Robert Koch Institute, 2025). These serovars also remain among the most common serovars in different animal and food products, despite having a relatively lower prevalence (EFSA and ECDC, 2023). *Salmonella* Derby and Infantis are commonly found in swine and poultry/poultry products, whereas *Salmonella* Typhimurium is frequently found in swine and swine products (Sévellec et al., 2020; Alvarez et al., 2023). *Salmonella* Enteritidis is commonly associated with contamination of eggs and poultry products. Nevertheless, these serovars might also occur in other food sources, such as bakery products or animal reservoirs, including cattle and beef, wild and domestic animals, or the environment (Munck et al., 2020), providing multiple routes of transmission to humans (Ferrari et al., 2019). Multiple research papers have emerged detailing the widespread occurrence and describing outbreak events linked to these predominant *Salmonella* serovars also across Germany in recent times (Methner, 2019; Simon et al., 2022; Sing, 2023).

The intensive use of antimicrobial agents in livestock has led to the emergence of multidrug-resistant (MDR) bacteria within the *Enterobacteriaceae* family (Mthembu et al., 2021). Animal sources, in particular, play a significant role in transmitting antimicrobial resistance isolates and determinants to human environments, that is, through the food chain (Perestrelo et al., 2022). Compared to other serovars, *Salmonella* Enteritidis has been reported to have a lower prevalence of antimicrobial resistance (AMR). However, a significant proportion of strains of serovars Typhimurium/1,4,[5],12:i:-, Derby, and Infantis found in livestock and food products are known to exhibit resistance to multiple antibiotic classes, including those commonly used in clinical settings (EFSA and ECDC, 2023). This can complicate treatment by limiting available options and increasing the risk of treatment failure in infected individuals. Especially the occurrence of bacterial isolates that are resistant to cephalosporins, fluoroquinolones, and carbapenems in animal production and food is a deeply concerning issue within the One Health approach (World Health Organization, 2019).

Cephalosporins are a widely used class of antibiotics with broad-spectrum activity against various bacterial pathogens (Chaudhry et al., 2019). Within the European Union, strict regulations have been implemented regarding their use, particularly for third- and fourth-generation cephalosporins in food-producing animals, as part of the strategy to curb antimicrobial resistance (Beber et al., 2025). In Germany, the overall use of cephalosporins has declined between 2017 and 2021 (Bonzelett et al., 2022). Despite this reduction, cephalosporins remain important in suckling piglets and, to a lesser extent, in dairy cattle, whereas in poultry their use is not licensed. The trend in resistance to cephalosporins has remained stable in Germany, but with a significant reduction in fattening pigs, and a general decrease has been observed across Europe (EFSA and ECDC, 2023; Flor and Tenhagen, 2025).

Cephalosporin resistance is often caused by the acquisition of resistance genes encoding extended-spectrum β-lactamase (ESBL) enzymes, conferring resistance also against third-generation cephalosporins. ESBL genes are typically located on mobile genetic elements, such as plasmids or transposons, facilitating horizontal gene transfer and contributing to the dissemination of cephalosporin resistance. CTX-M-type ESBLs have emerged as one of the most widespread ESBL variants globally. Remarkably, the dissemination of *bla*_CTX−M_ genes is facilitated significantly by the presence of IS*Ecp*1 transposons. These mobile genetic elements are commonly located upstream of the *bla*_CTX−M_ genes, promoting their mobility and expression within bacterial populations (Cantón et al., 2012; Davey et al., 2015; Bevan et al., 2017).

Studies on commensal *Escherichia coli* highlighted that the *bla*_CTX−M−15_ is generally the most prevalent ESBL gene identified in human isolates, partially linked to specific epidemic plasmids and clones, such as IncF multireplicon plasmids in *E. coli* of ST131, followed typically by *bla*_CTX−M−14_ (Mathers et al., 2015; Bevan et al., 2017; Carattoli et al., 2021; Mahmud et al., 2022). However, some studies also report *bla*_CTX−M−1_ as the second most frequent ESBL gene in human sources, especially in studies from Germany (Gerhold et al., 2016; Pietsch et al., 2017). In contrast, *bla*_CTX−M−1_ is the dominant ESBL gene found in livestock and food products in Germany and is generally associated with IncI1 plasmids, especially in animal sources (Irrgang et al., 2018; Carattoli et al., 2021). These studies highlight a complex global epidemiology of ESBL genes, with potential regional variations. A study on commensal food-derived *E. coli* in Germany underlined that the dissemination of the *bla*_CTX−M−1_ gene is mainly driven by their association with IS*Ecp*1 transposons located on IncI1 plasmids (Irrgang et al., 2018), as already described also in other countries across the world (Zurfluh et al., 2014; Tadesse et al., 2018; Valenzuela et al., 2023). For *S. enterica*, a recent study of human salmonellosis cases in Germany revealed that, in contrast to findings in *E. coli* from the human sector, the blaCTX-M-1 gene is the most prevalent ESBL gene (Pietsch et al., 2021).

Although *bla*_CTX−M−1_ is both the most common ESBL gene in commensal *E. coli* from livestock in Germany as well as the most common ESBL gene in *Salmonella* from the human sector in Germany, there is still a gap in knowledge on the occurrence and spread of the *bla*_CTX−M−1_ gene in *Salmonella* isolates from non-human sources in Germany. Therefore, this study aimed to expand knowledge and strengthen insight into the transmission of the *bla*_CTX−M−1_ gene in *Salmonella* isolates from isolates along the food production system in Germany. Here, we focused on serovars with the highest human health relevance in Germany and reported to harbor ESBL genes and examined their genetic variability as well as the variability of *bla*_CTX−M−1_ harboring mobile genetic elements, with *bla*_CTX−M−1_ integration sites in IncI1 plasmids emerging as the focal point of this study.

## Materials and methods

### Routine analysis in the NRL for *Salmonella* in Germany

The German National Reference Laboratory for *Salmonella* (NRL for *Salmonella*) annually receives 3,000–4,000 isolates from different non-human matrices (food, livestock, food production environment, and non-food-producing animals) across Germany. Isolates are routinely serotyped by slide agglutination with O- and H-antigen-specific sera (Sifin Diagnostics, Berlin, Germany) following the White–Kauffmann–Le Minor scheme.

Whole-genome sequencing has been routinely carried out since 2018 aiming a representative isolate selection, including isolates obtained within official controls (national monitoring, *Salmonella* control programs, official controls from competent authorities and self-monitoring controls from food business operators) as the minimal foundation, and added by isolates from further routine diagnostics, received or sequenced due to additional projects or in the scope of targeted outbreak analysis. Isolates are further selected by excluding repetitive isolates to avoid redundancy, where possible. All generated isolate WGS data undergo data processing and quality assessment using Aquaculture Information Management System (AQUAMIS), essential characterization using the in-house BakCharak Pipeline, and core genome multilocus sequence typing (cgMLST) analysis using Chewiesnake (see sections “Whole-genome sequencing and primary data analysis” and “Bioinformatics analysis”).

### Selection of isolates

The focus of this study was on *S. enterica* isolates belonging to *Salmonella* serovars Typhimurium (biphasic and monophasic Typhimurium 1,4 [5],12:i:-), Infantis, and Derby. All available *bla*_CTX−M−1_-positive isolate sequences from the NRL for the *Salmonella* WGS database were included in the study. To further expand this dataset, we retrospectively screened the MIC database (which includes all isolates from official controls, also including those from before 2018) for strains that showed phenotypic resistance to third-generation cephalosporins. Identified isolates were subjected to short-read whole-genome sequencing (see below), and further *bla*_CTX−M−1_-positive strains from a time period when WGS was not part of the NRL for *Salmonella* routine were included in the study.

With this approach, we aimed to include all existing *bla*_CTX−M−1_ positive isolates in the NRL collection obtained by official samplings as the data foundation, with added *bla*_CTX−M−1_ positive sequences available from other routine diagnostics (originating from livestock from other sampling approaches that are not laid down by law) to increase the amount of available data. Multi-sampling and genetically highly similar replicates have been excluded to reduce the data bias.

In total, the data set for this study resulted in 95 *bla*_CTX−M−1_-positive isolates (Typhimurium/1,4,[5],12:i:- (*n* = 34), Infantis (*n* = 22), and Derby (*n* = 39)). This selection represents *bla*_CTX−M−1_ positive *Salmonella* isolates from different years (2009–2022), different matrix categories (food, animal, and environment), different isolation sources (e.g., swine, cattle, poultry, and food derived thereof), as well as different German Federal States (see Supplementary Table S1), available at the German NRL.

Despite its human health relevance, serovar Enteritidis was excluded from the study because only one *bla*_CTX−M−1_ positive isolate was detected in the NRL for *Salmonella* sequence database.

### Selection of isolates for long-read sequencing

**Figure 1:**
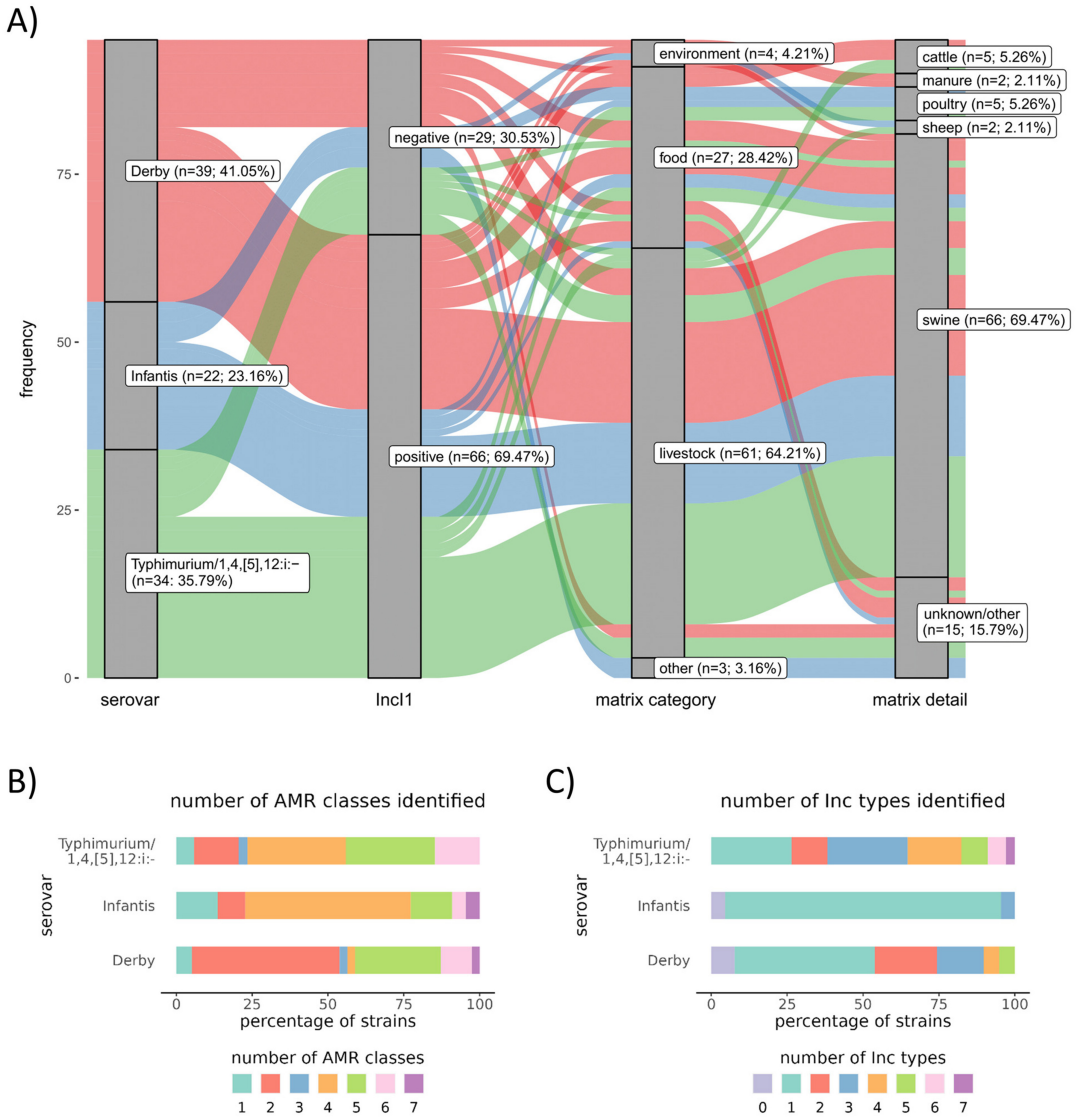
**(A)** Representation of 95 *bla*_CTX−M−1_-positive *Salmonella enterica* subsp. *enterica* isolates belonging to the serovars Derby (red), Infantis (blue), and Typhimurium/1,4,[5],12:i:- (green) across various reservoirs (food, environment, and livestock) and categorized by the presence or absence of the IncI1 plasmid marker. The visualization was generated using R version 4.2.2 with the “ggplot2” version 3.4.4 and “ggalluvial” version 0.12.5 packages and finalized in Inkscape version 0.92.4. **(B)** Illustration of resistance levels to different AMR classes dependent on the serovar as predicted by BakCharak. AMR classes included are Aminoglycosides, Beta-Lactams, Phenicol, Trimethoprim, Fosfomycin, Quinolone, Macrolide, Sulfonamide, and Tetracycline (see also Supplementary Figure S1). **(C)** Illustration of plasmid marker count dependent on the serovar as predicted by BakCharak (see also Supplementary Table S1).

Based on the genetic diversity of the 95 *bla*_CTX−M−1_ positive isolates in cgMLST analysis and BakCharak (see Bioinformatics analysis), their different geographical origins, year of isolation and their source of origin, a subset of *bla*_CTX−M−1_ positive isolates, representing a cross-section of *bla*_CTX−M−1_ harboring *Salmonella* circulating in Germany, was chosen and subjected to long-read sequencing (see section “Whole-genome sequencing and primary data analysis”). Hereby, we decided to focus on isolates that harbored an IncI1-plasmid (*n* = 66), since the combination of *bla*_CTX−M−1_ positive isolates with additional presence of an IncI1 plasmid was the most abundant one in our dataset (see Figure 1A). The selection process ended in a subset of 38 isolates [*S*. Derby (*n* = 12), *S*. Infantis (*n* = 13), and *S*. Typhimurium/1,4,[5],12:i:- (*n* = 13)] (see Supplementary Table S1).

### Strain cultivation and DNA extraction

*Salmonella* were cultivated on Luria Bertani (LB) agar, which, for retrospective isolates, was supplemented with 1 mg/L cefotaxime, and incubated overnight at 37 °C. A single colony was inoculated in liquid LB broth and cultivated under shaking conditions (180–220 rpm) at 37 °C for 14–16 h. Genomic DNA was extracted from liquid cultures using the PureLink^®^ Genomic DNA Mini Kit (Invitrogen, Carlsbad, CA, USA) according to the manufacturer's instructions.

### Whole-genome sequencing and primary data analysis

Libraries for Illumina short-read sequencing were prepared using either the Illumina Nextera XT or the Nextera DNA Flex/DNA Prep (M) Tagmentation kit (Illumina, San Diego, CA, USA, see Supplementary Table S2) according to the manufacturer's protocol. Paired-end sequencing was performed on Illumina MiSeq or Illumina NextSeq500 instruments using the MiSeq Reagent Kit version 3.0 (600 Cycles) or the NextSeq500/550 Mid Output Kit version 2.5 (300 Cycles), respectively. For short-read data quality control and assembly, the in-house pipeline AQUAMIS version 1.3.8 was used (Deneke et al., 2021a). This pipeline includes, amongst other programs, fastp version 0.22.0 (Chen et al., 2018) for quality assessment and trimming of short-reads, shovill version 1.1.0 (https://github.com/tseemann/shovill) for *de novo* assembly, QUality ASsessment Tool (QUAST) version 5.0.2 (Gurevich et al., 2013) for assembly QC, kraken2 version 2.1.2 (Gurevich et al., 2013) for taxonomic classification, and Confindr version 0.7.4 (Low et al., 2019) for detection of contamination.

Libraries for Oxford Nanopore Technologies (ONTs) long-read whole-genome sequencing were prepared using the Rapid Barcoding Kit 96 (SQK-RBK110.96, ONT, Oxford, UK) and sequenced for approximately 48 h on a MinIon Mk1C device using R.9.4.1 flow cells. Subsequently, fast5 raw data were transferred to a high-performance GPU computer, and basecalling using guppy version 6.0.1 in super-accurate (SUP) mode was performed. Long-read data was processed by the in-house pipeline MiLongA version 1.0.1 (https://gitlab.com/bfr_bioinformatics/milonga). ONT data were trimmed and filtered using Porechop version 0.2.4 (https://github.com/rrwick/Porechop) and NanoFilt version 2.8.0 (https://github.com/wdecoster/nanofilt) and quality checked using NanoStat version 1.5.0 (https://github.com/wdecoster/nanostat). *De novo* assembly was performed using Flye version 2.9 (Kolmogorov et al., 2019). Subsequently, the generated Flye assemblies were polished by Illumina short-read data using Pilon version 1.24 (Walker et al., 2014) in five iterations.

### Bioinformatics analysis

#### Bacterial characterization

For in-depth data analysis, the in-house pipeline, BakCharak version 3.0.4, for characterizing foodborne pathogens was used to confirm the serovar and to determine the seven-gene multilocus sequence typing (MLST) type, AMR genes, and plasmid markers (https://gitlab.com/bfr_bioinformatics/bakcharak). The BakCharak pipeline includes different modules for antimicrobial resistance (AMR) gene detection [tool: AMRfinder version 3.10.45; Database: National Center for Biotechnology Information (NCBI) resistance gene database (Feldgarden et al., 2019)], plasmid detection [tool: Abricate version 1.0.1 (https://github.com/tseemann/abricate); Database: CGE plasmidfinder (Carattoli et al., 2014)], and serotyping/seven-gene multilocus sequence typing (MLST) [tool: SISTR version 1.1.1; Database: SISTR (Yoshida et al., 2016)].

Sequence data for selected serovars were further analyzed with ChewieSnake version 3.2 (Deneke et al., 2021b) to perform core genome MLST (cgMLST) for hierarchical clustering of isolates per serovar.

#### Plasmid comparative analysis

IncI1 plasmid sequences were extracted from the Flye genome assemblies and annotated with Bakta version 1.4.0 (Schwengers et al., 2021) using Bakta's full version database version 5.0 (Schwengers, 2025) for downstream analysis.

To find closely related plasmids from other sources and geographical origins, we used the web service PLSDB (https://ccb-microbe.cs.uni-saarland.de/plsdb) version 2023-11-03_v2. This plasmid database includes 59,895 plasmid records. PLSDB gathers data from NCBI and the International Nucleotide Sequence Database Collaboration (INSDC), adding further filtering and annotation steps (Schmartz et al., 2022). Plasmids carrying the IncI1 marker and encoding the *bla*_CTX−M−1_ gene were downloaded, further characterized using BakCharak, annotated using Bakta, and incorporated for comparative analysis. Data regarding each plasmid's host strain MLST were obtained through literature research. For 10 isolates, no MLST data were available in the literature. In these cases, strain assemblies (*n* = 6) or raw reads (*n* = 4) were downloaded from NCBI, and data were processed as described in sections “Whole-genome sequencing and primary data analysis” and “Bacterial characterization” to obtain MLST data. For 20 plasmids, no information was found.

All 141 IncI1 plasmids (*n* = 38 of this study and *n* = 103 of the PLSDB database) were typed by plasmid multilocus sequence typing (pST) using the PubMLST database (Jolley et al., 2018). To decipher plasmid correlation, all plasmid sequences were aligned with MAFFT v7.750 and subjected to IQ-TREE2 version 2.2.6 (Minh et al., 2020) to infer a maximum likelihood tree. The automatic model selection option (ModelFinder) was used to estimate the best-fit substitution models, and branch estimation was assessed using the ultrafast bootstrap approximation UFBoot2 implemented in IQ-TREE2. Tree and metadata visualization were performed by means of iTOL (Letunic and Bork, 2021).

All plasmids were analyzed in-depth using Geneious Prime version 2023.2.1 (Biomatters, CA, USA) to identify and compare *bla*_CTX−M−1_ integration sites (ISts). Annotated plasmid sequences were manually analyzed, and comparative analysis was performed to establish similarities and a common plasmid backbone for each ISt.

### S1-based pulsed-field gel electrophoresis to confirm plasmid sizes obtained through long-read sequencing

Plasmid size determination was carried out using S1 nuclease-based (Thermo Fisher Scientific, Darmstadt, Germany) Pulsed-Field Gel Electrophoresis (PFGE) analysis utilizing the CHEF-DR III system (Bio-Rad Laboratories, Madrid, Spain) using standardized PulseNet protocol (https://www.pulsenetinternational.org/protocols/pfge; 2025-07-22) as previously described (Rodríguez et al., 2009). The XbaI (Thermo Fisher Scientific) digested *Salmonella* Braenderup strain H9812, or the Lambda Ladder (New England Biolabs, Ipswich, USA) served as a size marker. S1-PFGE analysis was performed on the 38 isolates selected for long-read sequencing to confirm the plasmid's presence and size.

## Results and discussion

### Characterization and spread of *bla*_*CTX*−*M*−1_ harboring *Salmonella* isolates in Germany

Of the 95 identified *bla*_CTX−M−1_ positive isolates, 64.2% (*n* = 61) originated from livestock and 28.4% (*n* = 27) from food sources, 4.2% (*n* = 4) from the environment, and 3.2% (*n* = 3) had an unknown origin. The majority of isolates are associated with swine (69.5%) (*n* = 66) and smaller portions are attributed to cattle (5.3%) (*n* = 5), poultry (5.3%) (*n* = 5), and sheep (2.1%) (*n* = 2) (Figure 1A). Moreover, the IncI1 plasmid replicon marker was the most frequently identified plasmid family marker across the three serovars in this dataset, accounting for 69.5% (*n* = 66) of all markers (Figure 1A). This is in line with previous findings that among the plasmid incompatibility (Inc) groups, IncI1 plasmids have been considered major vehicles for the dissemination of ESBL genes and, in particular, for the dissemination of *bla*_CTX−M−1_ from various sources (Carattoli et al., 2021).

Regarding additional resistance genes of the 95 *bla*_CTX−M−1_ positive isolates, a significant portion (>75%) of isolates belonging to the serovars Typhimurium/1,4,[5],12:i:- and Infantis, harbored resistance genes conferring resistance to more than three classes of antimicrobial agents (Figure 1B). Specifically, serovar Typhimurium/1,4,[5],12:i- isolates carried resistance genes against β-lactams (*bla*_TEM − 1_), aminoglycosides [*aph(3”)-Ib, aph(6)-Id*], and tetracyclines [*tet(B)*]. Serovar Infantis isolates harbored genes mediating resistance to trimethoprim (*dfrA17*) and aminoglycosides (*aadA5*). Resistance to sulfonamides through the *sul2* gene was found in isolates of both serovars. In contrast, the majority of isolates of *S*. Derby exhibited resistance genes against one or two AMR classes (Figure 1B). Notably, nearly all strains harbored the fosfomycin resistance gene *fosA7*. Most commonly detected resistance genes apart from *bla*_CTX−M−1_ were *sul2* (*n* = 57), *fosA7* (*n* = 37), *aph(3”)-Ib* (*n* = 34), *aph(6)-Id* (*n* = 34), *tet(B)* (*n* = 22), *aadA5* (*n* = 21), and *dfrA17* (*n* = 21) (see Supplementary Table S1 and Supplementary Figure S1).

When it comes to the detected plasmid Inc types, serovars Typhimurium/1,4,[5],12:i:- and Derby show a high diversity of different circulating plasmid types with up to seven or five plasmid Inc types within one isolate, respectively, while in *S*. Infantis maximum of three different plasmid markers per isolate could be detected (see Figure 1C). Among the different serovars, there are four strains without any plasmid and 36 strains harboring an IncI1 plasmid solely. Besides the IncI1 plasmid, various other plasmid markers were detected, including IncFIB, IncHI2, IncHI2A, IncFII, IncN, IncHI1A, IncHI1B, IncQ1, IncX1, and IncX3 and smaller ColE-like plasmids (see Supplementary Table S1 and Supplementary Figure S2). In this study, IncI1 plasmids are the most common plasmids in *bla*_CTX−M−1_ positive isolates and are frequently accompanied by other plasmid families, which is especially true for *S*. Typhimurium and *S*. Derby.

**Figure 2:**
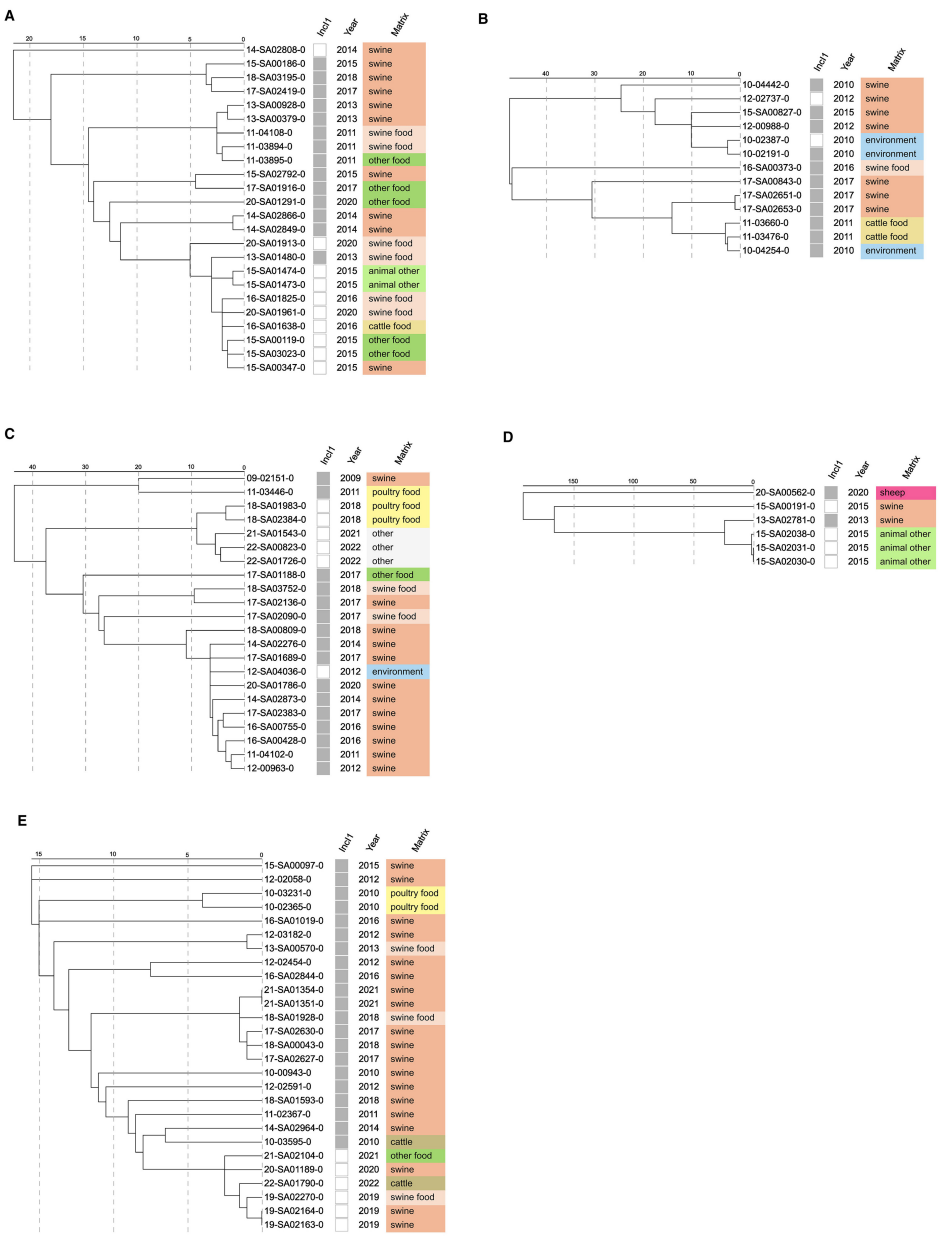
cgMLST analysis of *bla*_CTX−M−1_-positive *Salmonella enterica* isolates from the German strain collection of the NRL for *Salmonella*. The allelic distance matrix for each serovar and ST was hierarchically clustered using single-linkage clustering. Data regarding MLST, IncI1 plasmid presence (gray), year of isolation and source matrix are shown. **(A)** *S. Derby* ST39 isolates. **(B)** *S. Derby* ST40 isolates. **(C)** *S. Infantis* ST32 isolates. **(D)** *S. Typhimurium* biphasic ST19 isolates. **(E)** *S. Typhimurium* monophasic and biphasic ST34 isolates.

Hierarchical clustering based on cgMLST allele distance matrices of the *bla*_CTX−M−1_ positive isolates per serovar is depicted in Figures 2A–E. Isolates belonging to the same serovar often exhibit substantial genetic variability, as reflected by the allelic differences observed in isolates in this study. Genetic variability per serovar varied across MLST types per serovar. In serovar Derby, allelic distances ranged from 2 to 64 among ST39 isolates and from 2 to 140 among ST40 isolates. In serovar Infantis, ST32 isolates displayed allelic differences between 5 and 131. Similarly, within serovar Typhimurium, ST19 isolates differed by 0–422 alleles, while ST34 isolates showed a closer genetic range of 2–59 allelic differences. *S*. Derby isolates 11-00264-0 and 11-00347-0, both ST682, differed by 6 AD, and *S*. Typhimurium isolate 13-SA00799-0 (ST4081) are not depicted in Figure 2, due to their rare MLST type. In *S*. Infantis, isolates clustered by host species, with swine-derived isolates clustering together and poultry-derived isolates clustering separately (Figure 2C). This might be due to the spread of specific clonal *S*. Infantis lineages in the respective food production lines in Germany over the past decades. Similar results were obtained for *S*. Typhimurium, where isolates clustering tends to follow the host species (Figures 2D, E). Close genetic relation between *S*. Typhimurium ST34 isolates from swine and swine-derived food hints toward transmission of *bla*_CTX−M−1_ positive isolates along the food chain. Although our study is not designed as a prevalence study, detection of *bla*_CTX−M−1_ harboring *Salmonella* isolates, mainly in swine-derived isolates, is also in concordance with findings for commensal *E. coli* in the study of Perestrelo et al., where *bla*_CTX−M−1_ harboring isolates were described primarily originating from swine and cattle, whereas poultry is a minor reservoir (Perestrelo et al., 2022). These findings, moreover, potentially mirror the importance of the use of third and fourth generation cephalosporins in suckling piglets and partially dairy cattle in Germany and their restriction for use in poultry production.

The prevalence of *bla*_CTX−M−1_ genes in *E. coli* has already been widely studied, also in Germany, being mainly detected on animal farms (Fischer et al., 2014; Von Salviati et al., 2014), livestock and foodstuffs (Wu et al., 2013; Belmar Campos et al., 2014; Fischer et al., 2014; Day et al., 2016; Irrgang et al., 2018), but also in the environment (Savin et al., 2020), in hospitals (Pietsch et al., 2017) or in the community (Valenza et al., 2014), illustrating their varying importance in the different reservoirs. In *Salmonella*, *bla*_CXT − M−1_ is the most abundant ESBL gene in the human-sector clinical non-typhoid *Salmonella* isolates from Germany, with the main serovars affected being *S*. Infantis, *S*. Typhimurium, and *S*. Derby (Pietsch et al., 2021). Our dataset confirms the presence of *bla*_CTX−M−1_ in these *Salmonella* serovars, including isolates from non-human sources in Germany. This might reflect the nature of *Salmonella*, as a well-known foodborne bacterial pathogen, where the more animal-associated *bla*_CTX−M−1_ gene is co-transferred to human settings together with its *Salmonella* host through the food chain. *Salmonella* Enteritidis was excluded from this study, despite its utmost human health relevance, since the NRL strain collection revealed only one *bla*_CTX−M−1_ positive but IncI1 negative *S*. Enteritidis isolate (data not shown). This outcome indicates that this serovar is neither in the human sector in Germany (Pietsch et al., 2021) nor in humans and livestock at the European level, where it is associated with high AMR levels compared to other serovars (EFSA and ECDC, 2023).

However, in most of the studies on *bla*_CTX−M−1_ mentioned above, neither information on the localization of the *bla*_CTX−M−1_ gene nor its association with mobile genetic elements is given.

### *bla*_*CTX*−*M*−1_-associated IncI1 plasmids from *Salmonella*

Out of the 95 *bla*_CTX−M−1_ positive isolates selected in our study, 66 harbored an IncI1 plasmid (69.5%). This aligns with previous studies indicating that IncI1 plasmids are major vehicles for the dissemination of ESBL genes, particularly *bla*_CTX−M−1_, in *Salmonella* and related *Enterobacteriaceae* from various sources (Haenni et al., 2014; Wong et al., 2016). While IncF plasmids are widely recognized as the predominant carriers of ESBL genes in *E. coli* (Mahmud et al., 2022; Di Marcantonio et al., 2025), IncI1 plasmids appear to be the principal vectors of *bla*_CTX−M−1_ in *Salmonella*, especially in isolates from poultry and other animal origins (Wong et al., 2016; Carattoli et al., 2021). In particular, these findings support the most recent study on clinical *Salmonella* isolates from Germany, where IncI1 plasmids turned out to be the most prevalent *bla*_CTX−M−1_ harboring plasmid type, showing a high homogeneity in Derby, Enteritidis, Typhimurium, Stanley, and Brandenburg serovars (Pietsch et al., 2021). Also, this supports the from-fark-to-fork transmission of *Salmonella* isolates as a foodborne pathogen, where not only the plasmids are hosted, but also the plasmids and associated resistance genes are transferred. This contrasts findings in *E. coli* from human settings, where other CTX-M genes and plasmid family type combinations predominate, such as the well-described IncF–*bla*_CTX−M−15_ combination (Carattoli et al., 2021). In the 38 *bla*_CTX−M−1_ and IncI1 positive isolates (*S*. Derby, *n* = 12; Infantis, *n* = 13, and Typhimurium, *n* = 13) selected for in-depth analysis, the *bla*_CTX−M−1_ gene was confirmed to be located on the IncI1 plasmid. Long-read sequencing enabled the complete closure and circularization of these IncI1 plasmid sequences, which ranged in size from 82 to 111 kb. Since we confirmed the location of the *bla*_CTX−M−1_ gene in all 38 isolates of the in-depth study on the IncI1 plasmids, we infer that the *bla*_CTX−M−1_ gene is predominantly associated with IncI1 plasmids, also among the remaining 66 IncI1-positive isolates identified in this study. Nevertheless, this association was directly confirmed only for the subset of isolates analyzed by long-read sequencing, and thus, potential alternative locations of *bla*_CTX−M−1_, including chromosomal integration or linkage to other plasmid types, cannot be excluded. For the majority of isolates, the IncI1 plasmid sizes determined by S1-PFGE analysis mostly closely correspond to and confirm the size ranges established through long-read sequencing (data not shown). Plasmids were allocated in up to seven different plasmid sequence types (pSTs): pST3 (*n* = 16), pST58 (*n* = 7), pST49 (*n* = 5), pST7 (*n* = 4), pST26 (*n* = 1), pST63 (*n* = 1), and pST80 (*n* = 1). In three plasmids, the pST could not be determined.

**Figure 3:**
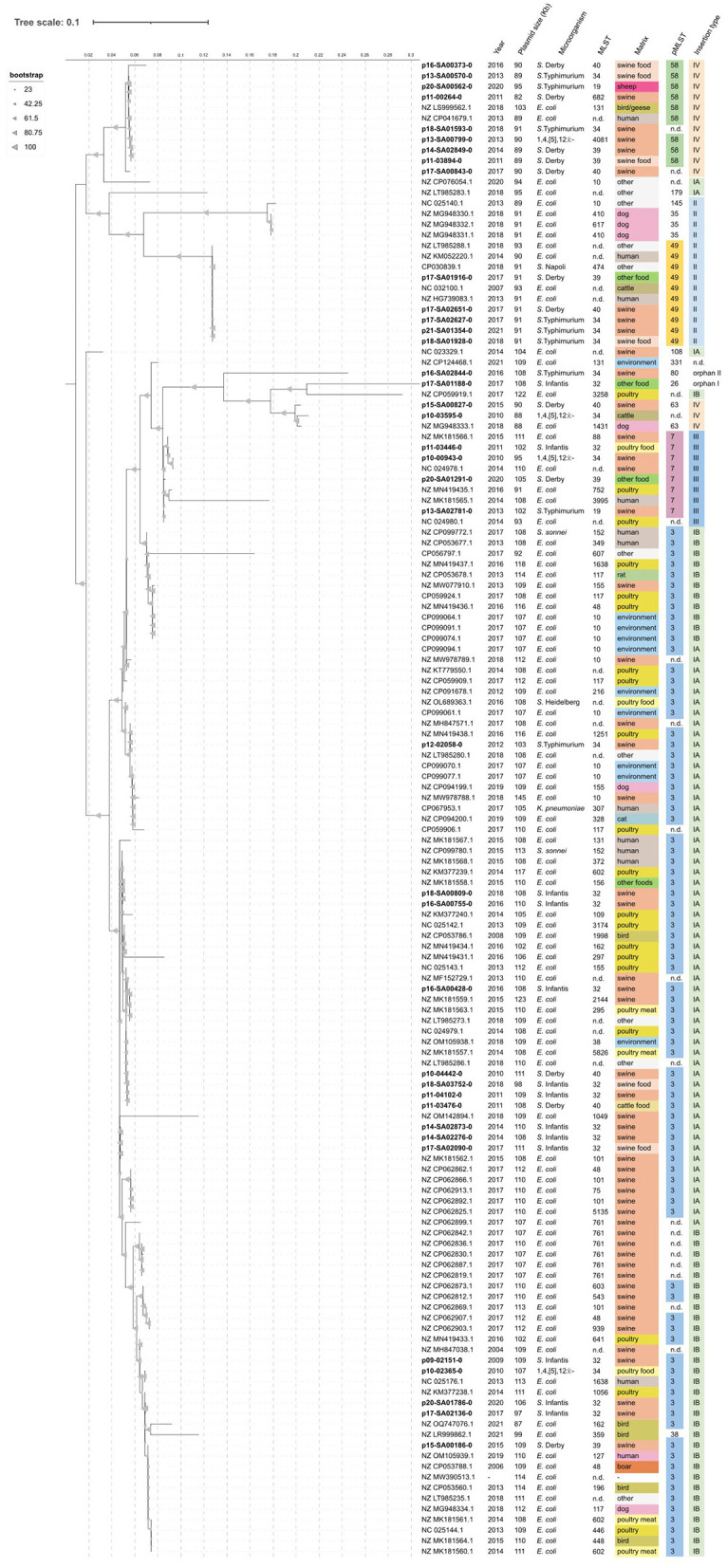
Phylogenetic clustering of selected IncI1 plasmids from this study and other sources (PLSDB database). Data regarding year of isolation, plasmid size, host microorganism, source matrix, pST, and detected integration site (ISt) for *bla*_CTX−M−1_ are shown. Bootstrap values are shown as percentages of 1,000 replicates and are represented by gray triangles on the three branches. Tree scale indicates the number of substitutions per site, and an internal scale system was added for additional stringency.

To reveal similarities of *Salmonella* IncI1 plasmids with other genera, especially from *E. coli* from different geographical origins and isolation sources, we used a comparison approach with plasmid sequences available through the PLSDB database. The search resulted in the identification of 103 similar IncI1 plasmids, all carrying the *bla*_CTX−M−1_ gene. Of the 103 plasmids analyzed, the vast majority (98/103) belonged to *E. coli*. The remaining five plasmids were distributed among other species: two from *Shigella sonnei*, and one each from *Klebsiella pneumoniae, S*. Napoli, and *S*. Heidelberg. Plasmids displayed pST3 (*n* = 67), pST49 (*n* = 5), pST7 (*n* = 4), pST35 (*n* = 3), pST58 (*n* = 2) and only one plasmid for pST38, pST63, pST108, pST145, pST179, and pST331 could be identified, whereas 16 plasmids could not be allocated to any pST (see Figure 3 and Supplementary Table S3).

pST3 was thus the most abundant plasmid type in both datasets. This is in line with findings of several studies carried out in humans and livestock that state the importance of pST3 and pST7 IncI1 plasmids in the transmission of AMR genes. In particular, studies on *bla*_CTX−M−1_ in *E. coli* show that pST3 IncI1 plasmids were more frequently associated with animal sources and less frequently found in humans. However, recent studies of large collections of human-origin ESBL *E. coli* isolates reveal a more diverse plasmid landscape, with other dominant ESBL types reflecting the evolving epidemiology in humans (Smith et al., 2015; Day et al., 2016; Zamudio et al., 2024).

The total of 141 plasmids, regardless of their pST, were subjected to phylogenetic analysis, clustering, and tree construction (Figure 3). *Salmonella* plasmid sequences from the NRL dataset in our study clustered according to their pST homogeneously with the selected 103 plasmids from the PLSDB. Neither the year of isolation, the geographical origin, the species, nor the *Salmonella* serovar appears to have an effect on plasmid phylogeny. Again, as in the case of the isolate's phylogeny, a trend in sub-clustering following matrix association can also be seen in cases of the plasmid phylogeny (Figure 3). These observations underline the capability of *bla*_CTX−M−1_ genes to spread horizontally among different bacterial hosts and reservoirs, mainly through their association with plasmids.

### IncI1 plasmids and associated AMR genes

Among the IncI1 plasmids analyzed in this study, we observed that several AMR genes only appeared in plasmids of certain plasmid sequence types (pSTs; Supplementary Table S3). For instance, 12 out of the 16 pST3 plasmids carried, in addition to the *bla*_CTX−M−1_ gene, a gene cassette harboring *dfrA17* and *aadA5* genes, conferring resistance against trimethoprim and aminoglycosides, respectively. This AMR profile is similar to those already reported in pST3 plasmids described for *E. coli* isolates from different livestock sources and countries (Abraham et al., 2018; Lucas et al., 2018). Comparable antimicrobial resistance profiles were reported in plasmids obtained from *E. coli* isolated from humans and chickens in Switzerland (Wang et al., 2014) and from chickens and swine in the Netherlands (Valcek et al., 2019). Moreover, in the study by Wang et al. (2014), the *sul2* gene was detected only in a single plasmid of human origin and was absent from chicken-derived plasmids, whereas Valcek et al. (2019) identified *sul2* in all plasmids analyzed.

Since AMR genes are frequently flanked by integration elements or associated with composite transposons, a close linkage between *bla*_CTX−M−1_ and specific surrounding genetic elements has already been described, such as ISCR1, IS10, IS26, and IS*Ecp*1 (Partridge et al., 2018). Of those, IS*Ecp*1 was most detected mobile genetic element (MGE) associated with *bla*_CTX−M−1_ in our study. This is in line with previous findings (Bevan et al., 2017). IS*Ecp*1–*bla*_CTX−M−1_ association has been detected in numerous studies conducted in Europe on *Enterobacteriaceae* isolates from various sources, displaying also a high prevalence in IncI1/pST3 plasmids (Hammerum et al., 2014; Jones-Dias et al., 2016; Lucas et al., 2018; Cormier et al., 2022). IS*Ecp*1 was previously identified upstream of *bla*_CTX−M−1_ and inserted into shufflon elements (Carattoli et al., 2021).

### *bla*_*CTX*−*M*−1_ gene integration sites in IncI1 plasmids

**Figure 4:**
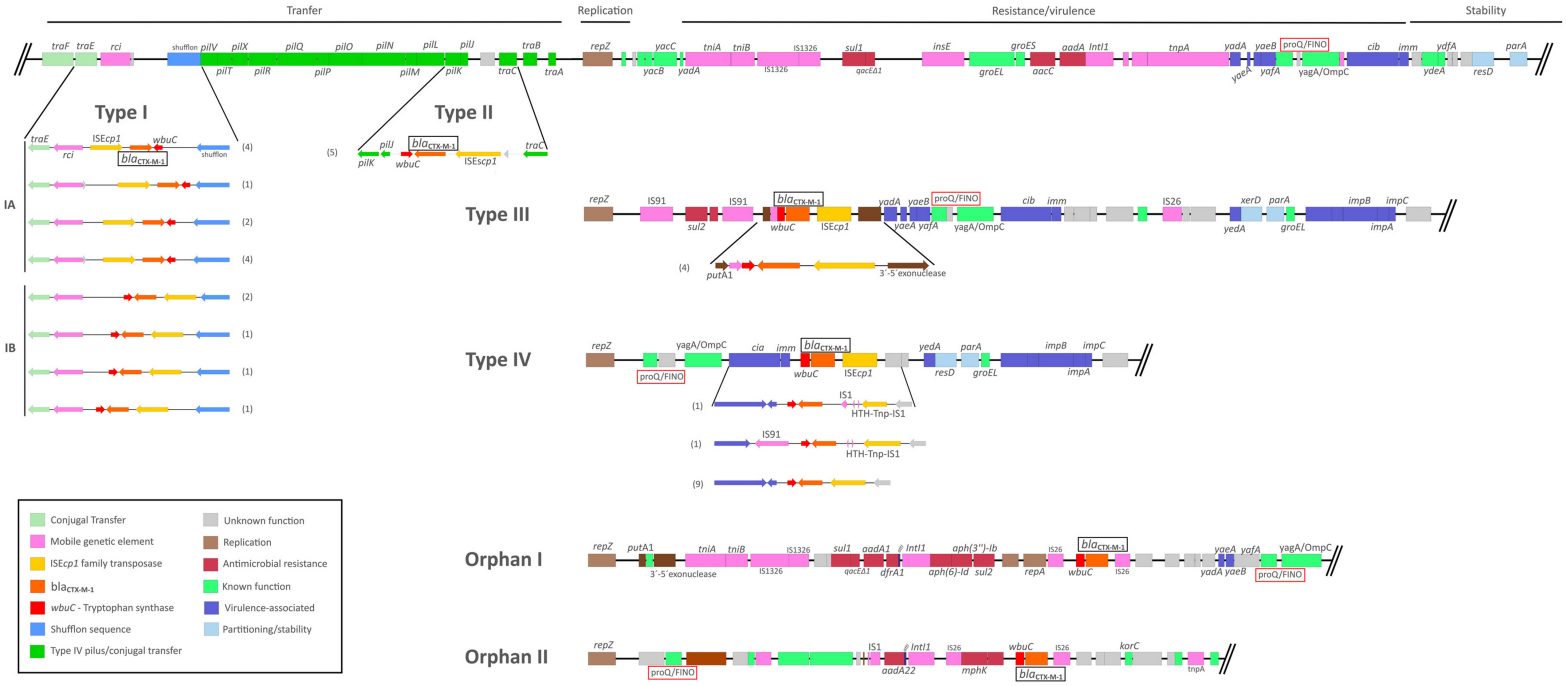
*bla*_CTX−M−1_ integration sites detected in the IncI1 plasmids from this study. IncI1 plasmid pSH1148_107 (Accession number AP005147) was used as the backbone of the figure plasmid. Regions were colored according to the study by Sampei et al. (2010). Regions include the conjugal transfer regions, plasmid replication and control regions, variable regions containing mobile genetic elements, antimicrobial resistance and/or virulence-associated genes, and genes associated with plasmid stability and partitioning. Numbers in brackets refer to counts of plasmids of each variant. Plasmids displaying ISt type I were previously described in Irrgang et al. (2018).

Genomic analysis of the *bla*_CTX−M−1_ localization gene on the IncI1 plasmids revealed six different integration sites (ISt) within the 38 isolates of this study. The detected ISts were named from “type I” to “type IV” based on the location of each ISt in regard to their distance to the origin of replication. Integration type orphans were named orphan I and orphan II, and were only detected in one plasmid each. ISt type I was found in the majority of the studied plasmids (*n* = 16), followed by ISt type IV (*n* = 11), ISt type II (*n* = 5), and ISt type III (*n* = 4) (see Figure 4). The PLSDB dataset was then screened for these ISt types, where only one further ISt could be observed, which was not detected in isolates from the NRL dataset (Figure 3).

#### ISt type I

ISt type I was identified in 16 plasmids of the study, which were all allocated to pST3. ISt type I was composed by the gene cassette IS*Ecp*1–*bla*_CTX−M−1_-*wbuC*, integrated in the IncI1 conjugative transfer region of the plasmid, between the genes for shufflon-specific recombinase and the shufflon proteins, or *pilV* gene for type IV pilus modification protein when the shufflon protein was lacking (Figure 4; Type I). The efficient conjugation system of the IncI1 plasmids is composed of a conjugative transfer region containing multiple inversion regions, known as shufflons, which aid the integration of foreign elements (Carattoli, 2013). Two variants of ISt type I, IA and IB, can be distinguished, depending on the orientation of the inserted element. Thus, ISt type IA was designated to the gene cassette flanked by *traE* and shufflon-specific recombinase genes upstream and shufflon proteins and *pilV* gene downstream. ISt type IB comprised the same flanking genes, but the cassette is inverted. Four different gene arrangements were detected for each ISt type I variant (Figure 4). The inverted versions of ISt type IA and IB were detected in 11 and 5 plasmids, respectively.

Eighty-five plasmids from the PLSDB database shared ISt type 1 with the NRL dataset, out of which 66 were allocated to pST3, only one plasmid each was allocated to pST38, pST108, and pST179. No pST was assigned to the remaining 16 plasmids associated with this ISt type. The inverted versions of ISt type IA and IB were detected in 46 and 39 plasmids, respectively. This ISt type 1 was already described by Irrgang et al. (2018), who investigated commensal *E. coli* isolates from food sources in Germany, mainly of poultry or cattle origin. In-depth analysis revealed the association of *bla*_CTX−M−1_ with IS*Ecp*1 elements on IncI1/pST3 plasmids for most isolates studied, albeit with the high genetic diversity among these isolates. Here, the gene cassette for IS*Ecp*1–*bla*_CTX−M−1_-*wbuC* was integrated in the IncI1 conjugative transfer region, between shufflon-specific recombinase and the *pil*V protein. The same two variants of integration (defined as IA and IB in our study), with six and seven subvariants each, were observed. Further in-depth analysis of the ISt types of several plasmids was carried out, originating from different bacterial hosts (*S. sonnei, K. pneumoniae, S*. Heidelberg, and mainly *E. coli*) and sources (poultry, swine, environment, domestic animals, and humans), described previously in different studies (Wang et al., 2014; Valcek et al., 2019; Mo et al., 2020; Poulin-Laprade et al., 2021; Matlock et al., 2022). These also revealed the aforementioned ISt type I variants, IA and IB (Figure 3).

#### ISt type II

ISt type II was identified in five plasmids in the NRL dataset, which were all allocated to pST49. ISt type II was composed of the same gene cassette as ISt type I, IS*Ecp*1–*bla*_CTX−M−1_-*wbuC*. However, ISt type II was located downstream in the IncI1 conjugative transfer region, between PilJ/pili assembly chaperones and a hypothetical protein near the conjugal transfer protein *tra*C gene.

ISt type II was identified in nine plasmids from the PLSDB database. Here, pST49 was the most abundant pST (*n* = 5), followed by pST35 (*n* = 3) and pST145 (*n* = 1). In-depth analysis of the ISt types in plasmids from similar studies revealed the same ISt type II. In Clément et al. (2018), the IncI1/pST49 plasmid isolated from *S*. Napoli from human origin shared the same pST and ISt type (CP030839.1). In the study by Jakobsen et al. (2015), they also identified IncI1/pST49 plasmids from *E. coli* in cattle and human feces, which we could assign to ISt type II (NC_032100.1 and NZ_KM052220.1). In a plasmid provided by Wang et al. (2014), originating from an *E. coli* isolate of human origin, the same ISt type II was detected, but in this case, IncI1/pST145 was observed (NC_025140.1).

#### ISt type III

ISt type III was identified in four of the NRL dataset IncI1 plasmids allocated to pST7. Similar to ISt type I and II, ISt type III was composed of the same gene cassette, IS*Ecp*1–*bla*_CTX−M−1_-*wbuC*. ISt type III is located behind the replication initiation gene *repZ*, between the *putA1* and 3′-5′ exonuclease genes (Figure 3). Mobile genetic elements and antimicrobial resistance genes appear upstream of the gene cassette, composed of IS91 elements and *sul2* genes for sulphonamide resistance. In ISt type III, the presence of fragmented IS91 elements indicates the occurrence of multiple overlapping integration events. IS91 fragmented regions of 1,209, 1,137, and 273 bp were detected in the virulence/resistance region of the plasmid, shorter than the 1,830 bp long IS91. Therefore, we suppose successive integration events have taken place: First, IS91-mediated incorporation of the AMR gene *sul2* and later, the integration of the *bla*_CTX−M−1_ gene mediated by the IS*Ecp*1 transposon. IS91 is widely reported to be involved in transmitting AMR genes in different pathogens, such as *Salmonella* (Lewis et al., 2023).

Five plasmids of the ISt type III were identified in the PLSDB dataset. All of them were assigned to pST7 as in isolates from our study, except for one for which no pST was determined. In-depth analysis of the ISt types revealed the same ISt type III in other studies' plasmids. In that way, ISt type III was identified in the IncI1/pST7 plasmid (NZ_MN419435.1) from a Norwegian study analyzing *E. coli* isolates from broiler flocks in Norway (Mo et al., 2020). Additionally, ISt type III was also detected in two IncI1/pST7 plasmids (NZ_MK181565.1 and NZ_MK181566.1) from the study of Valcek et al. (2019) isolated from Danish *E. coli* from swine and human, or IncI1/pST7 and IncI1/n.d. plasmids (NC_024978.1 and NC_024980.1) in the study of Brouwer et al. (2014) from *E. coli* from poultry and swine (Brouwer et al., 2014; Valcek et al., 2019). In contrast to the IncI1/pST7 plasmids from this study, the plasmid from Mo et al. (2020) lacked additional AMR genes, such as *sul2*, whereas this gene appeared in the other plasmids. Interestingly, one of the plasmids identified in our study (p20-SA01291-0) additionally carried *mef* (c) and *mph*(G) genes for macrolide resistance between the *sul2* gene and IS91 family transposase. This result underscores the persistent influence of flanking integration sequences on the integration or loss of AMR genes over time.

#### Orphan I

Orphan I was identified once in IncI1/pST26 plasmid p17-SA01188-0, from *S*. Infantis from swine. In this case, and unlike ISt types I-III, *bla*_CTX−M−1_ is flanked by IS26 (IS6 family) transposases, located in the virulence/resistance region of the orphan (Figure 4).

Although none of the plasmid sequences from PLSDB revealed this orphan 1 ISt, the appearance of IS26 is not unexpected, given that IS26 is one of the most commonly identified mobile elements associated with antibiotic resistance genes in both Gram-negative and Gram-positive bacterial species' plasmids and chromosomes (Partridge, 2011; Harmer and Hall, 2019). Indeed, IS26 and other members of the IS26 family are well-documented for their ability to create composite transposons and generate a multitude of rearrangements of multi-resistance regions due to recombination between inverted copies of IS26 (Harmer et al., 2020). This study also demonstrates that IS26 plays a pivotal role in the dissemination of antibiotic-resistance genes in Gram-negative bacteria. It is noteworthy to mention that the genetic elements of the plasmid are arranged similarly to the IncI1 plasmid pSH1148_107 (JN983049) isolated from *S*. Heidelberg, as described in Foley et al. (2021). Indeed, a BLAST comparison analysis for p17-SA01188-0 and pSH1148_107 plasmids revealed that both share a high global similarity, showing >99% sequence identity over 91% of the p17-SA01188-0 query sequence length. Plasmid lengths are similar but not coincident, ranging from 106,833 bp (pSH1148_107) to 108,310 bp (p17-SA01188-0), due to an 830 bp integration between *rci* and Shufflon A, and a variable region that spans the region downstream of the *sul1* resistance gene up to the IS26 transposon. Herein, we draw attention that p17-SA01188-0 and pSH1148_107 are very similar despite being isolated from different serovars and in different years, 2017 and 2011, respectively. However, some regions are dissimilar as a result of multiple integrations and deletions that may have occurred during that time span.

Furthermore, unlike other ISt detected in this study, only orphan I and ISt type III presented elements between the *repZ* gene and the proQ/FinO family protein gene, the latter involved in plasmid copy number control.

#### Orphan II

As orphan I, ISt orphan II was only identified once, in our NRL dataset in IncI1/pST80 plasmid p16-SA02844-0, from *S*. Typhimurium from swine. Similar to orphan I, the integration of the *bla*_CTX−M−1_ gene is mediated by an IS26 transposon and is located in the virulence/resistance region of the IncI1 plasmid. ISt orphan II is also comprised of a composite transposon driven by IS26, which embraces the *mphK* gene for macrolide phosphotransferase resistance. No orphan II ISt was detected in the PLSDB dataset.

#### ISt type IV

ISt type IV was identified in 11 plasmids from the NRL dataset belonging to pST58 (*n* = 7), pST63 (*n* = 1), or whose pST could not be determined (*n* = 3). Similar to ISt types I-III, the gene cassette was composed of IS*Ecp*1–*bla*_CTX−M−1_-*wbuC*. ISt type IV was characterized to be integrated between colicin-ia immunity protein Cia and two hypothetical proteins near the *yedA* gene. Further genomic analysis of the 11 plasmids was performed: up to three different variants of ISt type IV were detected due to the presence of additional IS elements. The first variant has no other IS than IS*Ecp*1 and was identified in nine out of 11 ISt type IV IncI1 plasmids. Nine plasmids categorized in ISt type IV have a high homology. This is also observed for ISt type IV/pST58 plasmid pESBL15 (CP041679.1) isolated by Bakkeren et al. (2019) from *E. coli* strain Z2115 from a clinical patient in Switzerland. For the two other ISt type IV classified IncI1 plasmids from the NRL dataset, IS1 or IS91 transposase integration was observed, constituting variants for this ISt type that were not previously reported (Figure 4).

Three plasmids from the PLSDB database showed the ISt type IV with pST63 (*n* = 1) and pST58 (*n* = 2). Among the studied plasmids, only one IncI1/pST331 plasmid from the PLSDB, NZ_CP124468.1, was not allocated to any ISt type considered here. However, it shares an inverted ISt type IV gene cassette. In addition, the location of the gene cassette interrupts the gene for colicin (*cia*).

Altogether, plasmid sequences clustered according to their pST, revealing a clear association between the plasmids' phylogeny and the identified ISt type (see Figure 3). Herein again, neither the origin, species, *Salmonella* serovars, nor the year of isolation appears to have an effect on plasmids clustering. In fact, each pST is associated with a certain ISt type, which suggests that the acquisition of the CTX-M-1 encoding genetic elements might have happened only a few times in ancestor plasmids. The successful spread of those plasmid lineages among different hosts and niches has facilitated the dissemination of the *bla*_CTX−M−1_ gene. Notably, none of the two datasets—neither the NRL dataset nor the PLSDB dataset—reflects a real prevalence situation, since both datasets underlie a data selection bias. Thus, the reasons for the rare occurrence of ISt types in single pSTs or orphan ISt types remain unclear but possibly be explained by still active transposition events of *bla*_CTX−M−1_ gene harboring elements or plasmid lineages that have not been spread as efficiently as the more common ones in the past. Also, ISt types found in more than one pST might indicate the various underlying alteration processes of plasmids, like rearrangements, co-integrate formation, and so forth.

## Conclusion

Our study focused on the occurrence of the *bla*_CTX−M−1_ gene in German *Salmonella* isolates from non-human sources in serovars of high relevance for human health in Germany. We reinforced evidence that IncI1 plasmids are a primary cause of *bla*_CTX−M−1_ gene transmission in *Salmonella* isolates from diverse geographical origins and along the food production line in Germany. Analysis of the integration sites of the *bla*_CTX−M−1_ gene in different IncI1 pSTs, together with the plasmids and isolates phylogeny, suggests that only a few independent integration events into IncI1 plasmids may have occurred in ancestor plasmids of different pSTs. This was supported by additional analysis of plasmid sequences obtained from the PLSDB database, including plasmids also from other genera such as *K. pneumoniae, S. sonnei*, and *E*. *coli*. Association of specific ISts of *bla*_CTX−M−1_ with certain plasmids pSTs was observed, with ISt IV/pST58, IStII/pST49, IStI/pST3, and ISt III/pST7 associations being prominent. Our results reflect that none of these combinations is restricted to *Salmonella* or a certain *Salmonella* serovar or to another genus and that the adaptive success of *bla*_CTX−M−1_ in *Salmonella* might depend mainly on the successful transmission of these four pST variants of IncI1 plasmids. Rare detections of the same ISts in other IncI1 pSTs beyond the main four listed above and orphan integration sites in the datasets of this study might reflect ongoing plasmid evolution or the presence of less successfully spread plasmid lineages. However, these hypothesis needs to be studied further.

## Data Availability

The original contributions presented in the study are included in the article/Supplementary material, further inquiries can be directed to the corresponding author/s.
