# Supplementary material for: Integration sites of blaCTX−M−1 relate to IncI1 plasmid phylogeny in Salmonella isolates from non-human sources in Germany
Source: Front Microbiol. 2026 Jan 21;16:1711391. doi: 10.3389/fmicb.2025.1711391 (PMC12868207; doi:10.3389/fmicb.2025.1711391)
Supplement: Supplementary file 1 [file Table_1.docx]

Supplementary Material


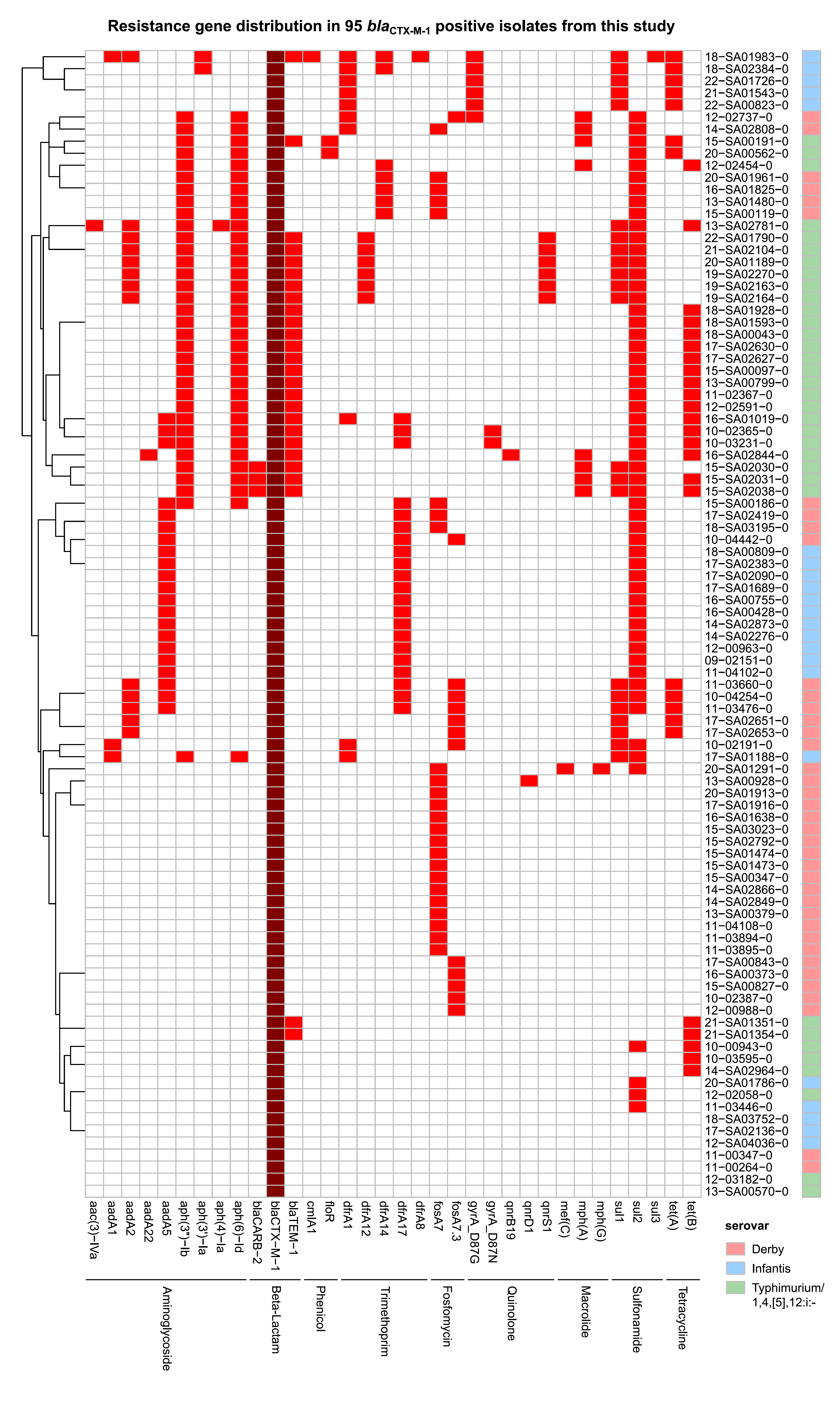


Figure S1: Absence and presence matrix illustrating the distribution of antimicrobial resistance (AMR) genes and AMR-related point mutations among the 95 isolates analyzed in this study. The prediction of the genes and point mutations was based on Illumina short-read assemblies processed by BakCharak v3.0.4. In BakCharak's methodology, an AMR gene is classified as 'present' when it meets the criteria for coverage (50 %) and identity. For identity, the curated thresholds of the AMRFinderPlus database were used, if no curated threshold exits, 90% were applied. Nonspecific genes categorized as AMR class 'EFFLUX' (*mdsA, mdsB*) found in all strains were excluded from the analysis. The figure was generated using R with the 'pheatmap' package and clustering of isolates was performed using the 'complete-linkage' method. The figure was finalized in Inkscape 0.92.4. The *bla*_CTX-M-1_ gene was highlighted in dark red.


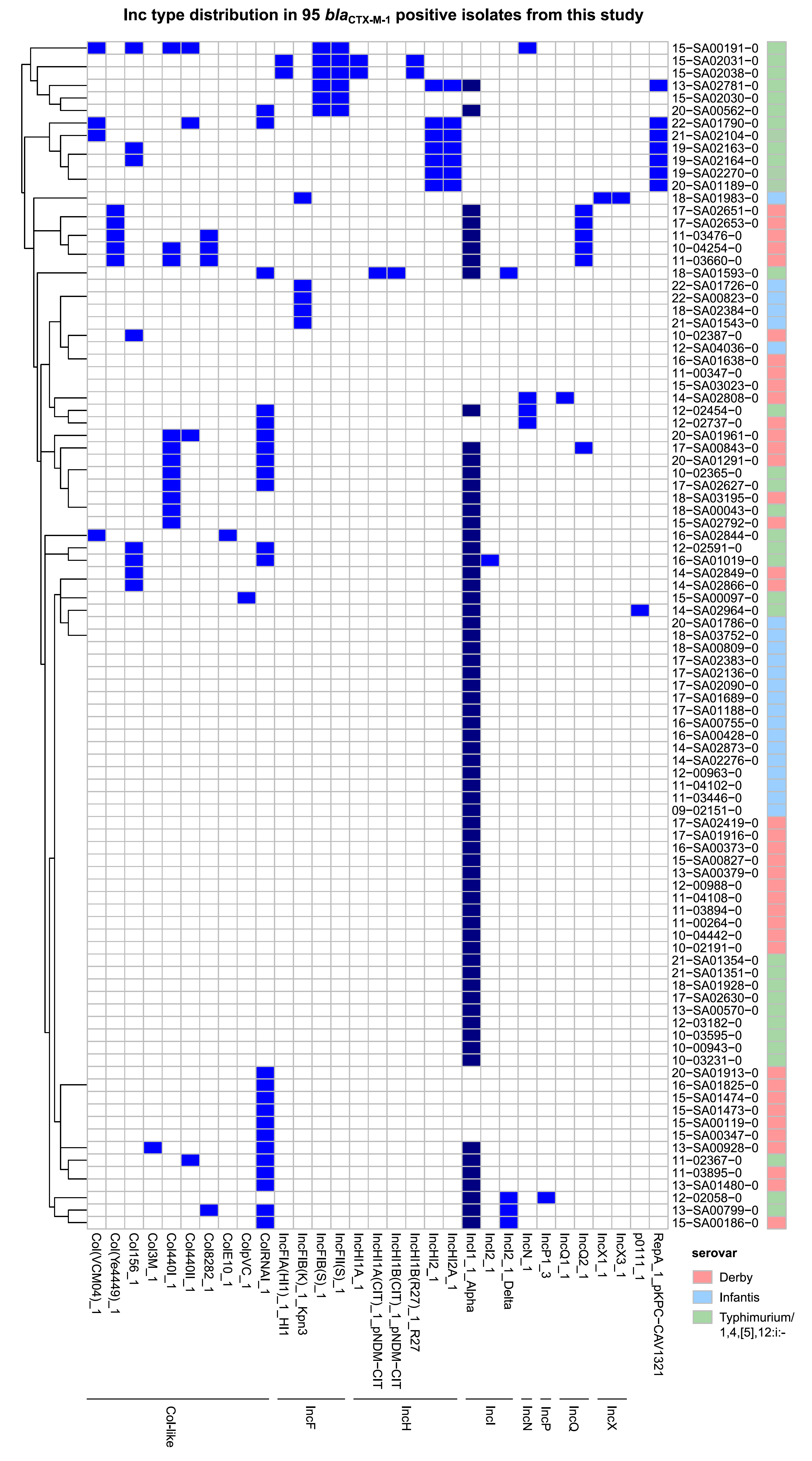


Figure S2: Absence and presence matrix illustrating the distribution of plasmid Inc types among the 95 isolates analyzed in this study. The prediction of the plasmid Inc types was based on Illumina short-read assemblies processed by BakCharak v3.0.4. In BakCharak's methodology, an Inc marker is classified as 'present' when it meets the criteria of at least 50% coverage and 80% identity. The figure was generated using R with the 'pheatmap' package and clustering of isolates was performed using the 'complete-linkage' method. The figure was finalized in Inkscape 0.92.4. The IncI1 plasmid marker was highlighted in dark blue.
